# Do people become more proactive at work as they grow older? Examining the mediating roles of intrinsic motivation, emotional exhaustion, and career aspiration

**DOI:** 10.3389/fpsyg.2023.1154861

**Published:** 2023-04-27

**Authors:** Wei Shi, Jean Fan Yang, Tianyi Sun, Yizhi Zeng, Zijun Cai

**Affiliations:** ^1^School of Labor and Human Resources, Renmin University of China, Beijing, China; ^2^Business School, Beijing Normal University, Beijing, China

**Keywords:** age, proactive work behavior, intrinsic motivation, career aspiration, emotional exhaustion

## Abstract

Today, there is an increasingly aging workforce. Previous studies have examined whether aged people exhibit more positive attitudes, better health, and better performance. However, the relationship between age and proactive work behavior has seldom been examined, which is unfortunate since organizations need employee proactivity to deal with uncertainty and unpredictability. Based on socioemotional selectivity theory, we propose that age might be positively related to proactive work behavior through intrinsic motivation and emotional exhaustion because older people tend to manage their emotions and obtain intrinsic enjoyment. But age might be negatively related to proactive work behavior through career aspiration because older people focus less on future development. With a sample of 393 people, we revealed intrinsic motivation and career aspiration. The findings could help us better understand how age is related to organizational outcomes and individual differences in proactive work behavior. They could also further reduce age-related discrimination and encourage organizations to manage older people in wise and better ways.

## Introduction

It has been widely acknowledged that the aging workforce has been a major organizational phenomenon that inspires much scholarly attention (North, 2019). In previous research, scholars found that age could influence various kinds of work-related outcomes, such as job performance (Alessandri et al., 2020), job attitudes (Dobrow et al., 2018), turnover intention (Ng and Feldman, 2009), innovation (Ng and Feldman, 2013a), health (Ng and Feldman, 2012), career satisfaction (Jung and Takeuchi, 2018), and leadership (Zacher et al., 2011). They generally got optimistic findings, showing that as people age, they would not necessarily experience low work effectiveness. These results have helped reduce age-related discrimination and encouraged organizations to manage older employees in better and wiser ways.

Despite these inspiring findings, the relationship between age and proactive work behavior has not been carefully examined. Proactive work behavior refers to the self-starting behavior to make constructive changes at work (Cai et al., 2019). As the business environment becomes more uncertain, this behavior has been regarded as the key to improving organizational competitiveness and individual effectiveness (Parker et al., 2019). To better understand the influence of the aging workforce on organizations, it is necessary to examine whether and why people display proactive work behavior as they age. Moreover, scholars have put much effort into examining who would be more proactive at work. They found that personalities, values, knowledge, and skills were significantly related to proactive work behavior (e.g., Zhang et al., 2016; Xu A. et al., 2022). To extend the understanding of individual differences in proactive work behavior, scholars have called for a life-span perspective, paying more attention to the role of age (Fay and Sonnentag, 2010). But this call has not received many responses in the last decade.

The current study aims to fill a blind spot by examining the underlying mechanisms linking age and proactive work behavior. Using the meta-analysis technic, Ng and Feldman (2012) found a positive association between age and proactive work behavior. They also found that age was not significantly related to role-breadth self-efficacy, an important mechanism linking distal factors and proactive work behavior (Hong et al., 2016). The findings urged scholars to explore other potential mechanisms. We extend these findings based on socioemotional selectivity theory (Rudolph et al., 2018). This theory focuses on the change in future time perspective during aging, arguing that as people age, they have a more constrained future time perspective. They would emphasize intrinsic and immediate enjoyment rather than future development. Accordingly, older people might better enjoy their work, feel more energized, and focus less on future career development. In this sense, age might have a positive association with proactive work behavior through intrinsic motivation and emotional exhaustion but a negative association with it through career aspiration.

By examining a multiple mediation model, the current study could make at least three contributions. First, we expanded the understanding of the influence of age at work. As we showed, age has a complex influence on whether people initiate changes at work. Second, we revealed how proactive work behavior was influenced by age. Previous studies mainly treated age as an unimportant control variable (e.g., Liu et al., 2022). But we showed that we should put age under the spotlight: It captures an important individual difference in proactivity at work. Third, we found a new mechanism: career aspiration. In the research about proactive work behaviors, previous studies mainly focused on work-related motivations (Cai et al., 2019), but we showed that the role of career-related attitudes should not be ignored.

## Hypotheses development

Aging is a complex process. People experience resource gain and loss simultaneously (Kanfer and Ackerman, 2004). For example, they may have better crystal intelligence and personalities, but at the same time experience a decrease in fluid intelligence and physical abilities (Ng and Feldman, 2013b). Among all the changes, socioemotional selectivity theory argues that the most important one is the change in future time perspective (Rudolph, 2016). This theory assumes that people have a fundamental awareness of time. As people age, their perception of time changes and they adjust their goals accordingly. Specifically, when people are young, they have a more open-ended future time perspective and perceive that they have enough time left. They prioritize long-term development. As they age, they have a more constrained future time perspective and perceive that they have a finite amount of time. They switch their goals to emphasize more immediate satisfaction. The change in goal priority influences their self-regulation, including motivation, decisions, and behaviors.

Proactive work behavior was influenced by different motivational states. As the proactive motivation model argues, three types of such states influence this behavior, namely “reason to,” which is about why people choose to be proactive, “can do,” which is about people’s confidence to conduct corresponding behavior, and “energized to,” which is about emotional states and general energy level to perform proactive work behavior (Parker et al., 2010; Cai et al., 2019). As Ng and Feldman (2012) showed, age was not significantly related to role-breadth self-efficacy, a typical “can do” factor. In the current study, we choose to focus on the other two types of motivational states.

Specifically, we argue that age may be positively related to intrinsic motivation, a typical “reason to” factor (Cai et al., 2019). According to socioemotional selectivity theory, age might be related to this factor through crafting their work and interpersonal relationships. As people age, they seek more immediate satisfaction. At work, younger people might endure factors that they do not like for future growth. However, older people might prefer the factors that provide meaning and enjoyment (Ng and Feldman, 2013b). For example, they were found to place greater importance on achievement, self-actualization, and autonomy at work (Kooij et al., 2011). They might search for jobs that provide these factors and even craft their jobs to fit their desires (Kooij, 2015; Kooij et al., 2017).

Moreover, older people tend to devote more energy to maintaining close relationships and building supportive networks (Ng and Feldman, 2013b). They were found to have fewer relationship conflicts, enjoy higher interpersonal trust, and receive more support from leaders (Ng and Feldman, 2010). Favorable interpersonal experiences are a critical source of intrinsic motivation (Deci et al., 2017).

With high intrinsic motivation, people tend to seek more challenges at work and want to do their work better. One way is to make constructive improvements by creating changes (Parker et al., 2010). Previous studies have consistently found a positive relationship between intrinsic motivation and proactive work behavior (e.g., Tu and Lu, 2013). Therefore, we propose:

*Hypothesis 1*: Age was positively related to proactive work behavior through intrinsic motivation.

As people age, they have a more finite future time perspective, which makes them put less importance on future development (Kooij et al., 2018). As a result, older people might have lower career aspirations, which reflects one’s career goals and captures one’s ambitions in career development (Napolitano et al., 2020). When people are young, they believe that they have much time left, so they have a more optimistic view about their future and set challenging career goals (Rudolph et al., 2018). As they age, they become aware that they no longer have enough time. They deemphasize future growth and have low motivation to learn and develop their careers (Ng and Feldman, 2012). As a result, they set less challenging career goals.

Career aspiration might also serve as a “reason to” factor that influences proactive work behavior because this behavior could also be seen as a career-oriented behavior (Cai et al., 2022b). By changing their work environment in constructive ways, people not only improve their performance at work but also send signals about their competence to others. Proactive people were found to gain higher status and visibility at work and have higher promotability (Weiss and Morrison, 2019; Xu A. J. et al., 2022). Those with high career aspirations would regard being proactive at work as a useful means to achieve their ambitions in their careers. Thus, career aspiration might help answer why some people are more proactive at work.

The above suggests that there might be a negative indirect effect through career aspiration. Note that this does not conflict with our previous hypothesis that there might be a positive indirect effect through intrinsic motivation. Intrinsic motivation reflects people’s enjoyment of their career work, but career aspiration reflects people’s future pursit. People might have high intrinsic motivation but low career aspirations. One example is when they enjoy their current jobs but do not want to take on additional responsibilities, which is usually required when people climb higher. They might also have low intrinsic motivation but high career aspirations. One example is when they see taking their current jobs as a necessary step to climb higher, although they do not like what they do not. In short, intrinsic motivation and career aspiration reflect different “reason to” states. Previous studies have shown that a variable can have contrasting indirect effects on an outcome variable (e.g., Cai et al., 2022a). Therefore, we propose:

*Hypothesis 2*: Age was negatively related to proactive work behavior through career aspiration.

Besides, older people are likely to have more positive emotional states at work (Scheibe et al., 2021). Socioemotional selectivity theory argues that when time is perceived to be limited, people tend to maximize positive affect and minimize negative affect. They maintain positive interpersonal relationships to keep them in positive moods. They regulate their feelings to keep them in positive states (Scheibe et al., 2016), so they were found to have higher emotional stability (Roberts et al., 2006) and stronger emotional regulation ability (Chapman and Hayslip, 2006). Facing difficulties, they adapt based on their resources and capacities to maintain effective functioning (Rudolph, 2016). Since positive emotional states are critical resources (Halbesleben et al., 2014), people with these states are less likely to feel emotionally exhausted. Previous studies have shown a negative relationship between them (e.g., Thompson et al., 2020). Besides, as we mentioned above, as people age, they put more importance on maintaining high-quality experiences, from which they could get social support. Since social support is also an important kind of resource (Halbesleben et al., 2014), through building meaningful interpersonal interactions, older people might be less exhausted at work.

Initiating change requires highly activated emotions and substantial energy (Bindl et al., 2012; Ouyang et al., 2019). When exhausted, people feel that their emotional tank is empty and are inclined to conserve their remaining energy (Wu et al., 2018). They would feel less “energized to” perform proactive work behavior (Cai et al., 2019). Therefore, we propose:

*Hypothesis 3*: Age was positively related to proactive work behavior through emotional exhaustion.

In short, based on socioemotional selectivity theory, we propose that age is related to proactive work behavior through three different mechanisms and the mediation effect of career aspiration might be in a reverse direction from those of the other two.

## Methods

### Data collection procedure

Data were collected from a large construction company in the northern part of China. After we told the head our research purpose, he allowed us to distribute surveys at this company and asked the HR department to help us. With the assistance of the HR department, we sent invitation messages to the employees *via* WeChat, a popular Chinese app. After receiving consent from the employees, we sent them survey links. Each participant has a unique survey ID so that we could match the data. The participants were told that they could quit at any time without any punishments and that their reported data would only be used for publication and would not be accessible to the management of the company. By confirming this information, we tried to ensure that they shared their true thoughts.

To alleviate the concern about common methods, we collected data at two time points with a one-week time lag. At time 1, we collected demographic information and mediators. At time 2, we collected the outcome variable. All data were self-reported. At time 1, 425 participants finished the survey. At time 2, 393 participants finished the survey. Thus we finally got 393 matched samples. Among the 393 participants, 290 were male. Two hundred and ninety had a bachelor’s degree and 90 had a high school degree. They were from different departments, such as construction, HR, security, and so forth, and doing different jobs, such as accountant, security check, worker, and so forth. Their average age was 35.71 years old (S.D. = 10.22 years old, maximum = 64 years old, minimum = 22 years old). Sixty-three of them were older than 50. Thus, we think the data were suitable to study the effect of age on proactive work behavior.

### Instruments

We used published instruments to measure our interested variables in this study. We adopted a 5-point Likert scale. Participants were asked to rate their agreements or frequency from 1 to 5, with 1 being the lowest and 5 being the highest. All the instruments have been used in the Chinese context. We thus followed the previous translation.

#### Intrinsic motivation

We used the scale developed by Gagné et al. (2015). Participants were asked to rate why they put effort into their current jobs, such as “because I have fun doing my job.” The Cronbach’s alpha in the current study was 0.90.

#### Emotional exhaustion

We used the 3-item scale developed by Watkins et al. (2015). Participants were asked to rate their agreement with the descriptions, such as “I feel emotionally drained from my work.” The Cronbach’s alpha in the current study was 0.80.

#### Career aspiration

We used the 5-item scale developed by Strauss et al. (2012). Participants were asked to rate their agreement with the descriptions, such as “I hope to become a leader in my career field.” The Cronbach’s alpha in the current study was 0.88.

#### Proactive work behavior

We used the 10-item scale developed by Parker and Collins (2010). Participants were asked to rate their frequency of displaying corresponding behavior at work, such as “generate creative ideas” and “try to bring about improved procedures.” The Cronbach’s alpha in the current study was 0.95.

## Results

### Confirmatory factor analysis

We first conducted a series of confirmatory factor analyses to examine the discriminative validity of the measures in the current study. The results are shown in Table 1. As we could see, the hypothesized 4-factor model fits the data better than other models (χ^2^ = 464, df = 180, CFI = 0.95, SRMR = 0.04, RMSEA = 0.06).

**Table 1:** Confirmatory factor analyses results.

Model	χ2	df	CFI	SRMR	RMSEA
Hypothesized 4-factor	464	180	0.95	0.04	0.06
3-factor (INT + EXH, ASP, PRO)	773	183	0.91	0.07	0.09
2-factor (INT + EXH + ASP, PRO)	1840	185	0.73	0.16	0.15
1-factor	3,011	189	0.55	0.16	0.20

INT, intrinsic motivation; EXH, emotional exhaustion; ASP, career aspiration; PRO, proactive work behavior. “+” Means that the variables were combined together.

### Descriptive statistics and simple correlations

The descriptive statistics and simple correlations are shown in Table 2. As we could see, age was correlated with intrinsic motivation (*r* = 0.15, *p* < 0.01), emotional exhaustion (*r* = −0.13, *p* < 0.05), and career aspiration (*r* = −0.15, *p* < 0.01) in an expected manner. Intrinsic motivation (*r* = 0.18, *p* < 0.001) and career aspiration (*r* = 0.29, *p* < 0.001) were also significantly correlated with proactive work behavior, but in different directions. Age was not significantly related to proactive work behavior (*r* = 0.08, *ns*), which might be because there are contrasting underlying mechanisms. We will test this in the following section.

**Table 2:** Descriptive statistics, reliability coefficients, and inter-correlations.

	Mean	SD	1	2	3	4	5
1. Age	35.71	10.2	/				
2. Intrinsic motivation	3.21	0.83	0.15**	**0.90**			
3. Emotional exhaustion	2.73	0.83	−0.13*	−0.40***	**0.80**		
4. Career aspiration	3.38	0.92	−0.15**	0.002	0.09	**0.88**	
5. Proactive work behavior	3.01	0.76	0.08	0.18***	−0.03	0.29***	**0.95**

They are reliability coefficients, i.e., Cronbach’s alpha coefficients, were shown along the diagonal. *p < 0.05. **p < 0.01. ***p < 0.001.

### Mediation analyses

**Figure 1:**
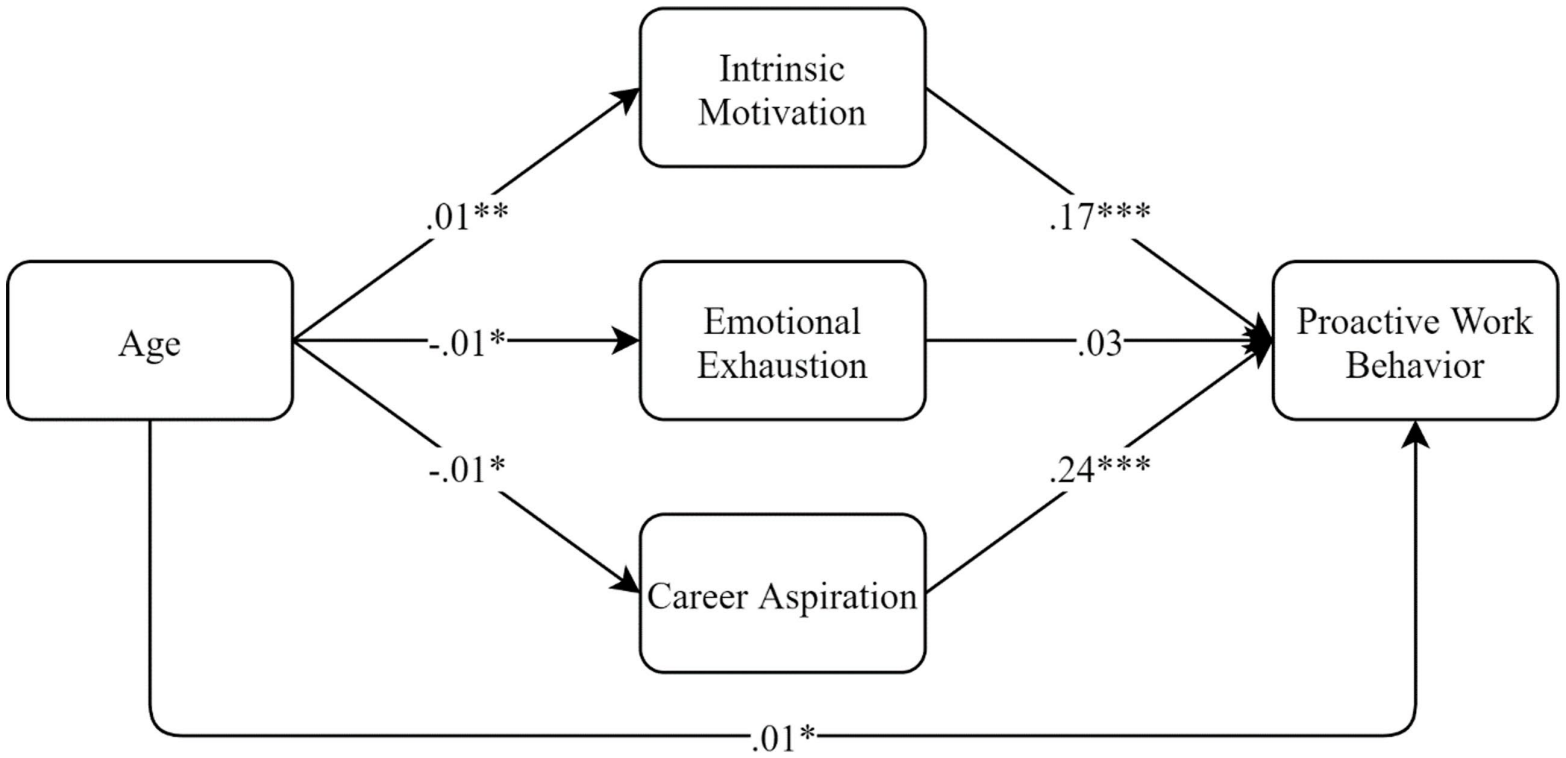
Mediation analyses results. *p < 0.05. **p < 0.01. ***p < 0.001.

We examined the mediation effects using PROCESS. Before analyses, independent and mediation variables were mean-centered. The results are shown in Figure 1. As we could see, age was significantly associated with intrinsic motivation (*β* = 0.01, *p* < 0.01), emotional exhaustion (*β* = −0.01, *p* < 0.01), and career aspiration (*β* = −0.01, *p* < 0.01). Intrinsic motivation (*β* = 0.17, *p* < 0.001) and career aspiration (*β* = 0.24, *p* < 0.001) were, in turn, significantly associated with proactive work behavior. We calculated the indirect effect with the bootstrapping method. The results showed that the effect through intrinsic motivation was significant: effect = 0.002, 95% CI = [0.001, 0.004]. That through career aspiration was also significant: effect = −0.003, 95% CI = [−0.006, −0.001]. However, the indirect effect through emotional exhaustion was not significant: effect = −0.0003, 95% CI = [−0.002, 0.001]. To note, after taking all the mediators into account, age was still significantly associated with proactive work behavior (*β* = 0.01, *p* < 0.05). Thus, we came to conclude that intrinsic motivation and career aspiration partially mediated the relationship between age and proactive work behavior.

## Discussion

This paper aims to examine whether and why age relates to proactive work behavior. Based on socioemotional selectivity theory, we propose intrinsic motivation, career aspiration, and emotional exhaustion to be three mediators. Results showed that age had contrasting effects on proactive work behavior through intrinsic motivation and career aspiration. Besides, age had a significant main effect even when the three mediators were included. The results contain important theoretical and practical implications.

## Theoretical implications

We first contribute to understanding the influence of age at work. Due to the aging workforce, management has put great effort into understanding the influence of age. In previous studies, they have examined the relationship between age with various outcomes (Truxillo et al., 2015). They also found that age could serve as a boundary factor that influences the effect of human resource management practices (Kooij et al., 2013), feedback (Wang et al., 2015), and so forth (Truxillo et al., 2015). Despite the rich findings, how age influences proactive work behavior has been largely ignored. Different from in-role behavior and citizenship behavior, proactive work behavior requires self-starting effort to create changes (Carpini et al., 2017). Thus, we could not simply apply previous findings to the relationship between age and proactive work behavior but need to examine it. Ng and Feldman (2012) revealed a positive simple association. Karanika-Murray (2022) found a moderator: cognitive demands. However, no one has so far examined the underlying mechanisms. We enrich existing understandings by revealing a double-edged effect: Age was positively related to proactive work behavior through intrinsic motivation, but was negatively related to it through career aspiration.

The results confirmed that age is significantly related to proactive work behavior and extend existing findings in several ways. We showed why there might be a positive relationship: due to intrinsic motivation. We also showed that we could not ignore the story on the other side: Age might have negative influences on proactive work behavior. By revealing the negative indirection effect through career aspiration, we could encourage future studies to take a more comprehensive view of the relationship through these two variables and to further explore potential moderators. The significant main effect indicates that there are other important mechanisms. For example, age differences in personalities and environments might influence proactive work behavior (Zacher and Kooij, 2017). In the future, scholars could further examine these possibilities.

The current study also extends the understanding of proactive work behavior. Individual differences in proactive work behavior have long been a hot topic. Previous research mainly focuses on personalities, such as the big five personalities (Wang et al., 2018; Tu et al., 2020), proactive personality (Fuller and Marler, 2009), and core self-evaluation (Aryee et al., 2017). Values and human capital have also been found to have important influences (Grant and Rothbard, 2013; Zhang et al., 2016). However, previous studies have ignored the age differences in proactive work behavior. As we showed, at different ages, people would display proactive work behavior at different frequencies. The findings correspond to the call of Fay and Sonnentag (2010) to more consider time in the research on employee proactivity. They suggest that besides the main effect, we could consider whether there are age differences in responding to the outer environment and personality effects. In the future, scholars could examine the moderation role of age.

Importantly, we revealed that age influences proactive work behavior through different mechanisms. While Parker et al. (2010) have proposed that proactive work behavior might be influenced by different motivational states, not many studies consider different mechanisms simultaneously (Hong et al., 2016; Wang et al., 2022). To do so is important because we could disentangle the mediation effects of different variables, compare their influences, and alleviate the concern for specious mediators (Peng et al., 2019). As we showed, when taking intrinsic motivation, emotional exhaustion, and career aspiration into consideration, the effect of emotional exhaustion became insignificant. There might be three reasons. First, compared with other mediators, emotional exhaustion was a weaker mediator. Second, the other two mediators could account for the relationship between emotional exhaustion and proactive work behavior. Third, there might be unexamined moderators. In the current study, the simple correlation between emotional exhaustion and proactive work behavior was not significant. In this sense, the third explanation seems to be stronger. In the future, scholars could try to replicate the findings with different antecedents and samples, to examine whether emotional exhaustion should be treated as a mediator.

We have heeded the call to take a more career-oriented perspective on proactive work behavior (Cai et al., 2022b). We showed that career aspiration, as a “reason to” factor, could mediate the relationship between age and proactive work behavior, even after taking intrinsic motivation and emotional exhaustion into consideration. Specifically, its effect size was similar to intrinsic motivation, a typical “reason to” state. This implies that at least for the relationship between age and proactive work behavior, career- and work-related thoughts and consideration play similar roles. In previous studies, scholars predominantly focused on work-related variables (Cai et al., 2019). However, advancing one’s career is an important part of one’s work life. Thus, the roles of career-related variables should not be ignored. Career adaptability and calling have been found to be significantly related to proactive work behavior (Cai et al., 2019; Cai W. et al., 2022). We add to this line of research by showing the significant role of career aspiration. That is, besides career-related capacity and orientation, career-related goals are also important factors. In the future, scholars could continue to examine the effects of other career-related variables, such as career motivation and confidence (Hirschi and Koen, 2021).

Last, we want to discuss the insignificant simple correlation between age and proactive work behavior. In this study, we aim to examine a multiple-mediation model. While traditional literature proposed that examining a mediation effect requires a significant correlation, scholars no longer hold this opinion (Edwards and Lambert, 2007; MacKinnon et al., 2012). There are many reasons. One reason is that the independent variable might be distal to the outcome variable, such that the effect size could be not captured by the simple correlation. For example, Greguras and Diefendorff (2010) did not find a significant simple correlation between proactive personality and in-role performance. However, they showed that it had significant indirect effects through two sequent mediators. One other reason is that there might be contrasting mechanisms so that the total effect turns out to be close to zero. For example, suppose that A indirectly influences C through only B1 and B2, and the indirect effects were, respectively, −0.01 and 0.01. Then, in theory, the simple correlation would be close to 0. The two reasons apply to our study. Age might be a distal predictor to proactive work behavior. It might influence psychological states through shaping personalities and environments (Zacher, 2015). Psychological states in turn influence the behavior. In this case, the simple correlation might not be strong. Besides, since there were contrasting mediation mechanisms with similar effect sizes, as we showed, the simple correlation, which represents the total effect between two variables, might be close to 0. In short, the insignificant simple correlation between age and proactive work behavior should not stop us from further examining the underlying mechanisms; rather, it should encourage us to think more carefully about the potentially complex mechanisms. We suggest scholars continue to explore other reasons why age might influence proactive work behavior.

## Practical implications

Findings from the current study should help further reduce the discrimination against older people at work and help organizations better manage these people. As we showed, older employees did not necessarily show less proactive work behavior. Although we found a null correlation, we showed that age was positively related to proactive work behavior through intrinsic motivation. In this sense, by altering management to fit older employees, organizations could motivate these employees to initiate changes at work. For example, management could design their work to fit older workers’ needs for supportive relationships (Truxillo et al., 2012). They could change their HR policies to help older employees fit into the work environment (Parker et al., 2020). Our findings also remind management to pay more attention to older employees’ career aspiration. As we showed, age was negatively related to proactive work behavior through career aspiration. Thus, management should care more about older workers’ career development and try to keep older workers ambitious about their left careers. Scholars have shown that enhancing career capacity and motivational orientations could help (Wang and Wanberg, 2017). In short, by unpacking the reasons why age influences proactive work behavior, we could provide theoretical foundations for organizational interventions and practices.

## Limitations

The current study suffers from several limitations. First, we adopted the self-reported method, which might introduce common method bias. As suggested by Podsakoff et al. (2003), we adopted a time-lag design to reduce the concern. We also made two *ad-hoc* analyses to check this possibility. First, we conducted a Harman one-factor test and found that the generated first factor only accounts for 36.44% of the total variances. Then, we added a latent method factor in confirmatory factor analyses. The iteration failed to converge. The results indicated that common method bias might not be a serious issue in this study (Fuller et al., 2016). In the future, scholars could ask peers of leaders to report proactive work behavior to further relieve the concern and adopt longitudinal designs to examine whether our results could be replicated. Second, we only include three possible mediators. However, there might be other related “reason to” and “energized to” factors. For example, positive affect might be a mediator. In the future, scholars could examine the effects of other possible mediators. Third, we did not examine moderation effects. According to socioemotional selectivity theory, job autonomy might be an important boundary factor. In the future, scholars could explore when would older workers be more proactive. Forth, although 16.03% of our participants were between age 50–59, which shows that there is a sufficient representation of older workers in our sample (Wang et al., 2015), we see that there are increasing focus and discussion about post retirement. Would our results change with an older sample become an interesting question. We thus encourage scholars to replicate our results with a different sample.

## Data availability statement

The raw data supporting the conclusions of this article will be made available by the authors, upon reasonable requests.

## Ethics statement

The studies involving human participants were reviewed and approved by Business School, Beijing Normal University. Written informed consent for participation was not required for this study in accordance with the national legislation and the institutional requirements.

## Author contributions

WS and ZC developed the idea and supervised the writing process. JY, TS, and YZ contributed to manuscript writing and data analyses. All authors contributed to the article and approved the submitted version.

## Conflict of interest

The authors declare that the research was conducted in the absence of any commercial or financial relationships that could be construed as a potential conflict of interest.

The reviewer QQ declared a shared affiliation with the authors WS and JY to the handling editor at the time of review.

## Publisher’s note

All claims expressed in this article are solely those of the authors and do not necessarily represent those of their affiliated organizations, or those of the publisher, the editors and the reviewers. Any product that may be evaluated in this article, or claim that may be made by its manufacturer, is not guaranteed or endorsed by the publisher.
